# Platelet Responses in Cardiovascular Disease: Sex-Related Differences in Nutritional and Pharmacological Interventions

**DOI:** 10.1155/2020/2342837

**Published:** 2020-05-19

**Authors:** Valeria Gasperi, M. Valeria Catani, Isabella Savini

**Affiliations:** Department of Experimental Medicine, Tor Vergata University of Rome, Rome 00133, Italy

## Abstract

Cardiovascular diseases (CVD) represent one of the biggest causes of death globally, and their prevalence, aetiology, and outcome are related to genetic, metabolic, and environmental factors, among which sex- and age-dependent differences may play a key role. Among CVD risk factors, platelet hyperactivity deserves particular mention, as it is involved in the pathophysiology of main cardiovascular events (including stroke, myocardial infarction, and peripheral vascular injury) and is closely related to sex/age differences. Several determinants (*e.g.*, hormonal status and traditional cardiovascular risk factors), together with platelet-related factors (*e.g.*, plasma membrane composition, receptor signaling, and platelet-derived microparticles) can elucidate sex-related disparity in platelet functionality and CVD onset and outcome, especially in relation to efficacy of current primary and secondary interventional strategies. Here, we examined the state of the art concerning sex differences in platelet biology and their relationship with specific cardiovascular events and responses to common antiplatelet therapies. Moreover, as healthy nutrition is widely recognized to play a key role in CVD, we also focused our attention on specific dietary components (especially polyunsaturated fatty acids and flavonoids) and patterns (such as Mediterranean diet), which also emerged to impact platelet functions in a sex-dependent manner. These results highlight that full understanding of gender-related differences will be useful for designing personalized strategies, in order to prevent and/or treat platelet-mediated vascular damage.

## 1. Introduction

Noncommunicable diseases (mostly, cardiovascular disease (CVD), cancer, diabetes, and chronic respiratory disease) are the leading cause of mortality worldwide: with 41 million deaths, they were responsible for 71% of all global deaths in 2016 [1]. Among them, CVD remains the biggest cause of death globally in the last 20 years: according to the last World Health Organization Report, ischemic heart disease and stroke collectively accounted for 15.2 million deaths in 2016 [1].

Genetic, metabolic, and environmental aspects interact together, leading to metabolic and/or physiological changes (overweight and obesity, rise in blood pressure, and increase in blood glucose and cholesterol levels), which represent key CVD risk factors. Some of the risk behaviours (tobacco use, physical inactivity, unhealthy diet, and alcohol abuse) can be deeply modified, in order to lower CVD prevalence.

Remarkably, CVD prevalence, aetiology, and outcome are also strictly related to differences in sex (based on biological characteristics, such as gene expression, hormones, anatomy, and immune system) and gender (based on social and structural determinants). Usually, life expectancy is greater for women than men, but CVD, including myocardial infarction, stroke, cardiomyopathy, and hypertensive heart disease, displays sex- and age-related incidence: indeed, it accounts for 40% of all deaths in men and up to 49% of all deaths in women over the age of 65 years [2]. This finding may be due to increased prevalence of CVD risk factors in women with respect to their male counterparts [3, 4]. In this context, it should also be recalled that CVD prevalence, outcome, prevention, and treatment in women are often underestimated, due to underrepresentation of women in CVD clinical trials [5, 6].

Among biological risk factors, platelets are emerging as new players, since increased platelet aggregation is a major determinant for heart attacks, stroke, and thrombosis: indeed, activated platelets are major components of thrombi occluding arteries and play a role in plaque formation within blood vessels during atherogenesis [7]. As a consequence, either antiplatelet therapy or other interventional strategies (such as those to promote consumption of foods rich in antioxidant and phytochemical compounds, fiber, and mono- and polyunsaturated fatty acids (PUFA)) are becoming increasingly relevant for preventing and treating vascular events in high-risk patients [8–16]. Accidentally, also in this context, a sex and gender disparity can be identified, in terms of aggregation response capacity and susceptibility to platelet aggregation inhibitors [17–19].

Based on this background, in this review, we will examine the state of the art concerning the main differences in platelet function between men and women, in order to establish their relationship with specific cardiovascular events and with responses to primary and secondary interventional strategies.

## 2. CVD Risk, Platelet Function, and Gender

Platelet count and mean platelet volume, usually linked to markers of platelet activation, are significantly associated with increased risk (as well as with outcome and mortality) of stroke, myocardial infarction, and coronary artery disease [20–22]. As a consequence, platelet number and size are commonly used tools for diagnosing and monitoring thromboembolic disorders [23, 24]. Activated platelets are also main players in atherogenesis, as they secrete proinflammatory chemokines that promote expression of adhesion molecules in atherosclerotic endothelial cells [25]. In atherosclerosis animal models, platelet/endothelium interaction precedes the onset of atherosclerotic lesions and inhibition of platelet adhesion decreases endothelium dysfunction and leucocyte recruitment in the atherosclerotic plaque [25]. Accordingly, currently used antiplatelet therapy has been proven to be effective in reducing thrombotic events (associated with a marked risk reduction of atherothrombotic events in high-risk settings, including patients with acute coronary syndromes), not only by inhibiting platelet activation but also by downregulating endothelial dysfunction and inflammation [26].

In 1999, Miller and collaborators highlighted that a gender-specific release of vasoactive factors from platelets could be found. In particular, they found that secretion of cyclooxygenase 1 (COX-1) metabolites of the *ω*-6 polyunsaturated fatty acid (PUFA) arachidonic acid (20 : 4 *ω*-6, AA) ((*i.e.*, thromboxane A2 (TxA2) and prostacyclin (PGI_2_)), as well as secretion of serotonin from platelets, was higher in male with respect to female pigs, but platelets from ovariectomized females had the highest concentration of all vasoactive compounds, with respect to male counterparts. This pioneering study highlighted, therefore, that sex differences in platelet activity might explain differences in response to injury in the coronary circulation, usually observed in males and females [27]. Since then, several studies have been undertaken to unravel sex-related dissimilarities and to understand how these differences can affect platelet biology and CVD onset.

It is well established that traditional CVD risk factors (including obesity, dyslipidaemia, inflammation, diabetes, hypertension, and smoking) are greater in women than in men and that CVD risks are more age-dependent in females than in males [28, 29]. Nonetheless, prevalence of CVD is greater in men than in women [30, 31]; this sex disparity persists until women reach menopause, when CVD incidence rapidly rises, until it overtakes that of men [32–35]. Accordingly, a study enrolling 59 men (58.6 ± 9.9 years) and 75 postmenopausal women (61.4 ± 10.6 years) with angina and nonobstructive coronary artery disease (ANOCA) found significant differences between the two groups in relation to lipid profile and thrombogenicity [36]. Indeed, females display higher levels of total cholesterol, total LDL-C, HDL-C and their subtypes and IDLs, as well as elevated thrombin-induced platelet-fibrin clot strength, clotting index, and fibrinogen activity. As no differences were seen on inflammatory markers (including OxLDL, OxLDL/*β*2GPI, and urinary 11-dehydrothromboxane B_2_), adverse cardiovascular events more frequently observed in females may be ascribed to the basal prothrombotic phenotype occurring in women with ANOCA [36–38].

The cardioprotective effects exerted by female hormonal levels produced during menstrual cycle (and lost after menopause) may explain the observed differences between sexes. Although the present review does not focus on molecular and cellular mechanisms of sex hormones, some details about their action (especially concerning platelets) need to be highlighted. Human platelets from both sexes express receptors for 17*β*-oestradiol (ER*α* and ER*β*) and ER-regulatory proteins, as well as androgen and progesterone receptors [39–42]. However, available literature data regarding interactions among sex, steroid hormones, and platelet functions are controversial. Indeed, either positive or negative and even no effects on platelet aggregation, in response to different agonists, have been reported in relation to sex [27, 43–47]. Additionally, a proaggregatory effect of 17*β*-oestradiol has been reported in both healthy male and female platelets [48]; on the contrary, Coleman et al.'s group [44] has recently reported that female platelets have both increased aggregation and activation potential, and 17*β*-oestradiol pretreatment feminizes male platelets leading to similar platelet behaviour in response to platelet-activating factor (PAF). Accordingly, as emerged by a large, population based, case-control study on risk factors for venous thrombosis (MEGA study), women taking oral contraceptives have a significant thromboembolic risk, with a positive association with estrogen dose used [49]. As recently reviewed, results concerning the impact of menopausal hormonal therapy on platelet activation in women are also largely contradictory, much likely due to heterogeneity in experimental settings among studies [44, 50]. This rather complex puzzle is further complicated by the evidence that testosterone can enhance TxA2 receptor density and platelet aggregability [51, 52].

Beside hormonal changes, other several factors related to platelet biology can account for the prothrombotic state; some of them, highlighted by several experimental and clinical evidence, will be described in the next paragraphs.

### 2.1. Platelet Fatty Acid Composition of Plasma Membrane

The main feature distinguishing male and female platelets is the fatty acid composition of plasma membrane, especially concerning *ω*-6 (AA) and *ω*-3 (eicosapentaenoic (20 : 5 *ω*-3, EPA) and docosahexaenoic (22 : 6 *ω*-3, DHA) acids) PUFAs. A study enrolling healthy 40 men and 34 women (age 20-50 years) of Caucasian origin showed that (albeit same daily income of these lipids, as well as *ω*-6/*ω*-3 PUFA ratio) women show greater incorporation of DHA and EPA into phosphatidylcholine and phosphatidylethanolamine, compared to men, who, on the contrary, show higher levels of AA and other *ω*-6 PUFAs [53]. Conversely, a controlled, randomized, double-blind study reported no significant sex differences in EPA and DHA incorporation into platelet membrane after 12-month supplementation with oily fish [54], although the too wide age range of subjects enrolled (*i.e.*, 20-90) might represent an important confounding factor not to be overlooked.

Higher *ω*-3 PUFA proportion in female phospholipids might be related to sex-dependent differences in conversion of the essential *α*-linolenic acid (18 : 3 *ω*-3, ALA) to EPA and DHA. Humans, indeed, can endogenously synthesize EPA and DHA from ALA through a series of desaturations and elongations, but young women show a better capacity to produce long-chain PUFAs which is dependent on estrogen effects [55]. In particular, in healthy young females, about 21% of dietary ALA is converted to EPA, and 9% is converted to DHA [56], whereas only 8% of dietary ALA is converted to EPA and 0-4% is converted to DHA in healthy young men [57]. As a consequence, women have greater circulating plasma DHA concentrations, thus increasing DHA supply for incorporation into platelet membranes [55, 58, 59]. Replacement of AA with EPA and/or DHA in female circulating platelets alters the phospholipid bilayer, thus modifying the activity of membrane-associated molecules (*i.e.*, phosphatidylserine, GPIIb/IIIa exposure, and P-selectin) [60, 61]. It also reduces generation of proaggregatory and proinflammatory eicosanoids, such as TxA2 (through competitive inhibition of COX-1) and 12-hydro(pero)xyeicosatetraenoic (12-H(P)ETE) acids (by competition for 12-lipoxygenase (12-LOX)) [62–64]. Consequently, the differential incorporation in membrane phospholipids leads to a different degree of platelet aggregation and vessel occlusion, thus contributing to the protective effects of *ω*-3 PUFAs on cardiovascular risk [64].

### 2.2. Platelets Receptors and Platelet-Derived Microparticles

Platelet receptors and downstream signaling cascades are affected by sex (and age), depending on the receptor engaged. A study carried out on healthy donors (53 men and 56 women; age range: 19-82 years in men and 21-70 years in women), indeed, showed sex- and aging-dependent decrease of platelet glycoprotein (GP) Ib/von Willebrand factor (vWF) interaction, with age-related changes more profound in women than in men [65]. Conversely, Sestito and coworkers [47] reported no changes in relation to sex (and age) in 62 healthy subjects (11 men and 17 women < 55 years and 22 men and 12 women > 55 years) in platelet response to collagen and ADP, although platelets from older men had higher tendency towards aggregability with respect to younger ones. Based on these findings, it should be considered that different experimental settings and sample stratification may explain different platelet behaviours.

Although no gender difference in total number of GPIIb-IIIa (fibrinogen receptor) was expressed on platelet surface, nonetheless, women show higher receptor reactivity: in response to ADP and thrombin receptor activating peptide (TRAP), indeed, the amount of fibrinogen/GPIIb-IIIa complexes was significantly greater in healthy women (especially in fertile subjects) than in men [66]. Sex-specific difference in GPIIb/IIIa activity also seems to emerge from the CRUSADE study, where female patients with non-ST-segment elevation acute coronary syndromes (NSTE ACS) and treated with GPIIb/IIIa antagonists (eptifibatide, tirofiban) showed more haemorrhagic events than males [67]. Although female gender is recognized as a risk factor for bleeding, especially following medical or surgery [68–70], nonetheless several confounding factors can be identified in the study, in particular, (i) difference in mean age (women: 65 ± 10 years, men: 60 ± 12 years), (ii) presence of other risk factors in women (obesity, hypertension) and, importantly, (iii) excessive dose administration in women compared to men could have biased results. Finally, it has been showed that platelets from males generally respond stronger to activation of the *α*2-adrenergic receptor by epinephrine and serotonin signaling pathways [71], while showing stronger TxA2 receptor-related aggregation responses [51].

During activation, platelets release microparticles (pMPs), a heterogeneous population of membrane vesicles with distinct functional properties: based on their cargo of molecules and antigenic composition, indeed, these pMPs can modulate several biological functions, such as coagulation, inflammation, and transfer of bioactive molecules to other cells [72–74]. High prothrombotic activity has extensively been reported for circulating pMPs [75, 76], which can be considered specific candidate biomarkers for CVD diagnosis and prognosis in early and late disease processes. Indeed, plasma pMP levels are high in healthy subjects showing high coronary heart disease risk score [77], and their number and phenotype (*i.e.*, surface expression of P-selectin and phosphatidylserine) positively correlate with recurrent CVD events [78, 79].

According to their parental origin, a significant gender-specific difference has been found, with the amount of pMPs significantly greater in healthy women than in the corresponding counterparts [80]. A menstrual cycle-dependent difference in pMPs also exists: a case-control study enrolling 27 healthy women and 18 healthy men demonstrated increased pMP release in females, especially in the luteal phase [81]. This finding suggests, therefore, that higher circulating pMPs (together with other risk factors, including pregnancy, oral contraceptives, and hormone therapy) may contribute to higher risk of developing venous thromboembolism observed in women < 45 years [49, 82, 83].

### 2.3. Platelet and CVD Risk in Metabolic Syndrome

Metabolic syndrome is a cluster of cardiometabolic risk factors, including central obesity, hypertension, impaired glucose metabolism, and dyslipidaemia [84, 85]. Several meta-analyses have shown that CVD risk in metabolic syndrome is higher in females than in males and sex differences in adiposity and insulin resistance may partly account for this increased risk [86, 87]. A Korean cross-sectional study, carried on 3827 participants (2169 men and 1658 women), showed a positive correlation for platelet count and an inverse correlation for mean platelet volume in women with metabolic syndrome, but such trend was not observed in men [88]. Moreover, a recent prospective longitudinal, observational, cohort study (the Framingham Heart Study) evaluated protein biomarker profiles in 3289 men and 3895 women (mean age 49 years), in order to identify key biological pathways differing between sexes [89]. Of 71 biomarkers analyzed, 86% were significantly different in the two groups; in particular, women showed upregulation of pathways involved in inflammation, immune response, and adiposity, while platelet homeostasis and fibrosis pathways were enriched in men [89]. According to available literature data, these sex differences in circulating CVD biomarkers were attenuated in postmenopausal women, confirming that CVD risk dramatically increases following menopause [89]. Given these gender-related divergences, physicians are encouraged to take care of sex-specific risks in primary cardiovascular prevention and for design of personalized therapeutic strategies [90].

## 3. Intervention Studies

Several interventional strategies have been established to reduce incidence of clinical events related to coronary heart, cerebrovascular, and peripheral vascular diseases. World guidelines are drawn up for primary (for lowering risk in people without clinical symptoms) and secondary (for people with clinically manifest CVD) prevention, and concern both lifestyle changes and drug use. Herein, we will focus on dietary habits and antiplatelet therapy since these are the interventions where gender differences are most evident.

### 3.1. Nutritional Strategies

Until few years ago, in studies concerning correlations between nutrition and CVD, the traditional research orientation was to identify harmful foods (e.g., unprocessed red and processed meats, sugar-sweetened beverages) and nutrients (such as *trans*- and saturated fats, cholesterol, and sodium), whose consumption is now strictly prohibited [15, 91]. As it is recently emerging, instead, the importance of the so-called “positive food/nutrients,” whose diet reduction or absence plays an equally crucial role in increasing cardiovascular risk [15, 91]. Coherently, diets based on foods particularly rich in antioxidants, phytochemicals, fiber, vitamins, monounsaturated fatty acids, and PUFAs, such as Mediterranean diet (MD) and vegetarian diet, are recognized worldwide as protective against CVD and its risk factors [8–15]. Also, in this context, it has been recognized a sex-related difference in individual responses to specific dietary habits (*e.g.*, adherence to MD), with men displaying more favorable specific cardiometabolic changes, with respect to premenopausal women [92–95].

Some food patterns and bioactive components appear particularly interesting, as, besides their beneficial cardiovascular effects, they also act on platelets with a sex-depending impact.

#### 3.1.1. Mediterranean Diet

MD, a typical eating pattern of the Mediterranean basin, is characterized by wide consumption of fruits, vegetables, cereals, legumes, fish, olive oil as main fat source, and moderate red wine intake. Due to the consumption of these food items, subjects adhering to MD assume significant amounts of main nutrients of a healthy diet [96].

Effects of MD adherence on platelets have been investigated in the Moli-sani population-based cohort study that enrolled 14586 Italian healthy men and women [97]. Food intake was determined by food frequency questionnaire, while adherence to the MD was analyzed by using the MD Score (MDS) that evaluates intake of specific MD items and the Italian Mediterranean Index (IMI), a score particularly related to typical products consumed in Italy. What emerged from this study is that (i) greater adherence to MD was significantly associated with reduction in platelet count; (ii) subjects with greater adherence had reduced odds of being in the highest platelet count group; and (iii) hypercholesterolemia and increase in C-reactive protein (marker of inflammation) were prevalent in high-platelet count individuals. However, the most intriguing finding was that, although the mean platelet count of all individuals was within normal-range values, nonetheless, it directly correlates with predicted CVD risks in men, but not in women [97]. In line with this finding, a previous Moli-sani population-based cohort study demonstrated that specific healthy MD foods, particularly rich in antioxidant and phytochemicals, protected men much more than women against hypertension and inflammation [96]. This finding, therefore, suggests that healthy dietetic habits represent a valid strategy for primary CVD prevention that, however, requires particular attention to sex-related responses.

#### 3.1.2. *ω*-3 PUFA

Beyond different efficiency of *ω*-3 PUFA biosynthesis in men and women, the rate of conversion is however low to satisfy physiological needs [55–57]; therefore, nutritional guidelines recommend to take EPA and DHA from fish (particularly, cold-water fatty fish, such as salmon, mackerel, tuna, herring, and sardine fish) and other seafood. Sometimes, also EPA and DHA supplementation, in the form of oily fish or fish oil (often fish liver oil) or krill oil capsules, is advised, although content of EPA and DHA varies in each of these preparations [98].

Several population studies have shown that dietary fish intake (as part of a healthy eating pattern) is inversely associated with stroke incidence and mortality, and therefore, EPA and DHA are counted among those nutrients that benefit cardiovascular health [99, 100]. As emerged from epidemiological studies, among mechanisms underlying the EPA and/or DHA cardiovascular protective role, reduction of platelet activation deserves special mention. The Reduction of Cardiovascular Events with Icosapent Ethyl-Intervention Trial (REDUCE-IT) study, where over 8000 high-risk patients were enrolled and followed for five years, demonstrated that supplementation with EPA-ethyl ester (providing more EPA per gram of oil, with respect to other supplements) significantly reduced major CVD events by 25% [101]. The beneficial effect much likely derives from the ability of EPA to compete with AA as a substrate for platelet COX-1, thus counterbalancing production of proaggregatory thromboxane A2 [102].

The main finding emerging from different studies is that *ω*-3 PUFAs counterbalance CVD risk factors in a gender-specific manner, both in primary and secondary prevention [103]. A meta-analysis indicated that dietary intake of fish and *ω*-3 PUFAs was correlated with lower incidence and mortality of stroke, especially in women and those with body mass index (BMI) < 24 kg/m^2^ [104]. Phang and collaborators [105] have shown that 1.0 *μ*M EPA, DHA, and docosapentaenoic acid (DPA) reduced aggregation of platelets isolated from healthy subjects. When data from male and female populations were combined together, according to already published literature [106–108], all tested *ω*-3 PUFAs reduced platelet aggregation, but with significant differences in terms of efficaciousness: EPA resulted to be the most effective showing a significantly higher percentage inhibition with respect to both DHA and DPA. When data were separated by sex, the same pattern of inhibition of platelet aggregation by *ω*-3 PUFAs was observed only in the male group, while differences were lost in females. The most pronounced antiaggregatory effect of EPA observed only in men might, therefore, suggest a positive interaction between sex hormones and *ω*-3 PUFAs in modulating platelet activation cascades [105]. The same group [109] confirmed these findings also *in vivo*: in a blinded placebo-controlled intervention trial (enrolling 15 male and 15 female participants), the effects of a single acute oral dose of EPA- (providing 1 g EPA; EPA/DHA ratio = 5 : 1) or DHA- (providing 1 g DHA; EPA/DHA ratio = 1 : 5) rich oils on the aggregation response were investigated. Again, a gender-specific response was seen: DHA, but not EPA, significantly reduced platelet aggregation in women, whereas EPA, but not DHA, exerted an inhibitory action only in men. Accordingly, an inverse relationship between testosterone levels and platelet aggregation following EPA supplementation was observed. What emerges is, therefore, that males may benefit more from EPA supplementation and this may partly be explained considering that, among w3-PUFAs, EPA is more efficiently incorporated into male platelets [59]. Moreover, the finding that females are more responsive to DHA seems to be coherent with the evidence that, independently of dietary intake, females have higher circulating DHA concentrations, compared to males [59]. In the same population, authors also observed a gender-dependent response in the procoagulant activity of circulating pMPs. In male subjects, the single dose of EPA-rich oil inhibits pMP activity (-22%), in parallel with inhibition of platelet aggregation; on the contrary, DHA-rich oil reduces platelet aggregation, independently of pMP activity, in female subjects [110], thus pointing out to pMPs as one of the potential mechanistic pathways whereby *ω*-3 PUFAs might differentially modulate platelet activity and, therefore, yield cardiovascular benefits.

However, the study has some limitations, including differences in baseline characteristics (females were older and of postmenopausal age, while males had greater BMI and high testosterone levels) and in platelet-related parameters (longer lag time in males; higher platelet count and baseline platelet aggregation in females).

Thus, although available evidence highlights gender-specific effects of *ω*-3 PUFAs on platelet function, further work is needed to establish exact mechanisms underlying the interactions between sex hormones and this class of nutrients and future well-powered studies should be assessed to justify dietary recommendations for distinctive *ω*-3 PUFAs in men and women.

Although there are several data on beneficial effects of *ω*-3 PUFAs (taken from fish or supplements) in high-risk CVD patients, nonetheless, their therapeutic value, up to now, is not clear, as results are not conclusive and sometimes controversial [101]. Moreover, several confounding, often perplexing, factors should be considered, such as (i) differences in taking PUFAs from fish (which also is a source of other important nutrients, like selenium, iodine, zinc, calcium, and proteins), fortified foods (*e.g.*, enriched margarine), or supplements; (ii) harmful effects related to high-PUFA intake, especially through supplements, *i.e.*, high concentrations of toxic compounds (namely, mercury, dioxins, and polychlorinated biphenyls) in fish oils; (iii) other negative events dependent on *ω*-3 PUFAs themselves, such as prolonged bleeding time, increased lipid peroxidation, and abrogation of normal immune responses.

Although dietary modifications may help in preventing pathological conditions, all these elements point out that we are far from a solid, scientific-based knowledge for development of individualized PUFA supplementation regimens to prevent and manage CVD, and further studies are required to better define precise dietary indications.

#### 3.1.3. Flavonoids

Flavonoids are a large family of over 5,000 hydroxylated polyphenolic compounds, which encompass six major subclasses of dietary significance, named anthocyanidins, flavan-3-ols (also referred as flavanols), flavonols, flavanones, flavones, and isoflavones. These phytochemicals are abundantly found in fruits, vegetables, and cocoa, as well as in beverages, such as tea and wine. Several factors may affect flavonoid content in food, among which are agricultural practices, environmental conditions, ripening, storage, and food processing; consequently, reported value contents in plants should be considered approximate [54, 111].

Flavonoids are often present as glycosides (such as isoflavones and anthocyanins), and this chemical feature, together with other factors (including some other chemical characteristics, interactions with other components of food matrix, composition of colonic microbiota, and gut and liver metabolism), influences their metabolic fate and bioavailability [54, 111]. For example, anthocyanins and galloylated catechins are poorly absorbed, while isoflavones seem to be the most bioavailable flavonoids [111].

Beyond these evidences, flavonoids have received particular attention for potential health benefits of fruit- and vegetable-rich diets, especially in relation to the cardiovascular system [111]. Most (but not all) epidemiological studies, indeed, greatly suggest that high intake of dietary flavonoids (approximately 200 mg/day of total flavonoids) is inversely related to CVD risk and mortality [112–117]. Nonetheless, it must not be overlooked that some of their beneficial effects may also be attributed to other bioactive constituents, (including other polyphenols, vitamins, and minerals), synergizing with flavonoids.

If initially flavonoids were believed to mainly act as antioxidants, nowadays, it is well established that they positively impact cardiovascular health by exerting other biological activities, such as (i) induction of vascular endothelium relaxation, (ii) inhibition of endothelial dysfunction, (iii) stimulation of nitric oxide release, (iv) inhibition of platelet aggregation, and (vi) downregulation of proinflammatory mediators [116, 118]. There is, indeed, the consistent view that these compounds directly act on various signaling pathways, among them, those related to P2Y_1_/P2Y_12_ (ADP receptors), GPVI (collagen receptor), protease-activated receptor 1 (PAR1; thrombin receptor), and COX-1 signaling, through which flavonoids (especially, those extracted from cocoa, tea, pigmented rice, chokeberry, and oat) mitigate platelet adhesion, degranulation, and aggregation [119].

In this context, interventional studies have shown that flavanols, which include catechin, epicatechin gallate, epigallocatechin, and epigallocatechin gallate monomers; their dimers (theaflavins, thearubigins); and polymers (proanthocyanidins), appear the most efficacious in attenuating platelet hyperactivation. Most of the studies are focused on two of principal dietary sources of flavanols, *i.e.*, cocoa-based products and green tea [120]. Just an example, a double-blind randomized placebo-controlled trial, enrolling twenty patients with congestive heart failure, evaluated acute and chronic effects of commercially available flavanol-rich chocolate on platelet and endothelial functions and compared it with a chocolate-free cocoa liquor, as control. The authors reported that, shortly after ingestion, only flavanol-rich cocoa led to peripheral vasodilatation, endothelial function improvement, and reduction in platelet activation [121]. The exact contribution of flavanols in the beneficial effect of cocoa has further been assessed by Ostertag and coworkers [122], who evaluated potential sex differences in platelet responses. In their blinded randomized, controlled trial, the researchers compared flavanol-enriched dark chocolate (containing 907.4 ± 22.75 mg of flavan-3-ols) with both standard dark (containing 382.3 ± 45.20 mg of flavanols) and white (with no flavanols) chocolates, in relation to effects on platelets derived from healthy men and women. By pooling data from male and female groups, they found that acute consumption of the two types of dark chocolates reduced ADP- and thrombin-dependent activation and aggregation of platelets and increased the collagen/epinephrine-induced *ex vivo* bleeding time; these effects were time-dependent and more evident with flavanol-enriched dark chocolate. According to gender-related differences in platelet signaling cascades [51, 66, 71], ADP-triggered pathways were significantly inhibited in men, while thrombin-dependent signaling was exclusively attenuated in women, after consumption of flavanol-enriched dark chocolate. Analysis of plasma or urine concentrations of flavanols and their metabolites revealed gender-related absorption or metabolism of flavanols that might partially explain the different efficacy by which these phytochemicals can affect platelet functions [122]. However, it should be underlined that also white chocolate improved platelet profile in males [122], thus indicating the presence of other compounds, not yet identified, capable of exerting antiplatelet effects in a sex-dependent manner.

Besides flavanols, isoflavones (such as daidzein and genistein) deserve to be mentioned. These flavonoids, mainly found in soybeans and soy foods, show both estrogenic and antiestrogenic effects; therefore, they are also classified as phytoestrogens [111]. Accordingly, the effects derived from their intake (*via* foods and supplements) are object of extensive investigations, especially in the hormone-sensitive cancer research field. Moreover, it is well known that their assumption ameliorates some symptoms of menopause, such as hot flashes [123]. This evidence, together with the finding that isoflavones ameliorate lipid profile and endothelial function in a gender-specific manner, strengthens the idea that these phytoestrogens are also beneficial for cardiovascular health [124–126], especially for menopausal women, missing estrogen-dependent protection. For example, three prospective cohort studies have found positive correlation between higher intake of isoflavones and tofu (but not soy drinks) and moderately lower risk of developing coronary heart disease in both men and women; nonetheless, in women, the favorable association of tofu was more pronounced in young subjects or postmenopausal women without hormone use [127].

The capability of isoflavones to inhibit *in vitro* platelet activation induced by collagen or AA, through a mechanism dependent on their ability to act as TxA2 receptor antagonists, seems noteworthy [128]. A double-blind, randomized study has clearly underlined that supplementation with soy-derived isoflavones reduced the risk of thrombogenesis, by decreasing platelet TxA2 signaling [129]. Twenty-nine healthy postmenopausal women (aged 45-60 years) were randomly assigned to two groups, receiving either 100 mg/day soy isoflavone extract or placebo, for 3 months; what emerged is that supplementation had no significant effect on common CVD risk factors (lipid profile, blood pressure, and anthropometric measures), while significantly decreasing TxA2 receptor density (from 181.9 ± 30.9 to 115.2 ± 16.2 fmol/10^8^ platelets) [129]. Conversely, a previous study evaluating the chronic effect of soy protein supplements (that are rich in isoflavones) in healthy young males showed that, although soy supplementation critically increased plasma concentration of isoflavones, nonetheless, such increase was not sufficient either to significantly inhibit *ex vivo* platelet aggregation or to ameliorate lipid profile [130].

In conclusion, although the impact of diet and gender on platelets is suggestive (Table 1), dietary manipulation of platelet function is still far from being realized, since gaps in our knowledge (especially concerning sex differences on bioavailability, metabolism, and activity of food components) persist and more research is required.

## 4. Antiplatelet Therapy

Current antiplatelet therapies mainly target enzymes (such as COX-1), receptors (such as thromboxane or ADP receptors), and glycoproteins (such as GPIb or GPVI) [132, 133]. Among antiplatelet drugs, the most widely used is aspirin that irreversibly inhibits COX-1, thus preventing conversion of AA into TxA2; nonetheless, it does not act on TxA2-independent signaling pathways and, moreover, long-term usage leads to increased risk of bleeding events [134]. To overcome these limitations, other drugs have been developed, such as the P2Y12 receptor inhibitors clopidogrel, prasugrel and ticagrelor. The first one is the most commonly used, but it shows a delayed therapeutic onset and may cause coagulation dysfunction; prasugrel inhibits platelet aggregation more rapidly than clopidogrel; the last antiplatelet drug exerts CVD protective effects without increasing overall bleeding and, being a P2Y12 receptor reversible inhibitor, loses pharmacological activity upon body clearance [135, 136].

Although women are less represented in cardiovascular clinical trials, nonetheless, numerous investigations have pointed out that some of the abovementioned female-related conditions (such as hormonal status and platelet biology) have to be taken into account in view of aspirin administration for primary prevention. Just an example, the Women's Health Study, evaluating the efficacy of aspirin in 39876 healthy women (≥45 years of age) monitored for 10 years, reported a significant prevention of ischemic stroke (RR = 0.83; *P* < 0.04); however, the drug also led to a parallel increment of gastrointestinal bleeding risk [137]. Other clinical trials and meta-analyses confirmed no significant benefit of aspirin treatment in women concerning cardiovascular events and CVD mortality, but a huge increase in risk of overall bleeding [134, 138]. Based on these findings, special attention should be paid when treating women with aspirin.

A meta-analysis of five randomized trials, involving 79,613 (of whom 23,533 are women) patients with cardiovascular heart disease, showed that clopidogrel (in addition to aspirin) significantly decreased cardiovascular events in both men and women. Although gender differences in the absolute benefit are not striking, during long-term antiplatelet therapy, risk of events was higher in women than in men and clopidogrel therapy seemed to be effective only in men. As documented by the *post hoc* subanalysis of the BleeMACS study, collecting data from fifteen centres in Europe (Germany, Greece, Italy, Netherlands, Poland, and Spain), Asia (China and Japan), North America (Canada), and South America (Brazil), the increased bleeding rates observed only in females were associated with prasugrel-/ticagrelor-based dual antiplatelet therapy [139]. Finally, the multicentre, Italian START ANTIPLATELET registry investigated the choice of antiplatelet treatment and its impact on one-year clinical outcome, in 625 males and 215 females presenting with acute coronary syndrome. In this study, what emerged is that dual antiplatelet therapy was more commonly prescribed in men and, when administered in both sexes, clopidogrel was the best choice for women, while prasugrel was preferentially used in men. However, gender-related differences in terms of therapy did not lead to different outcomes. Therefore, P2Y12 inhibitor choice in dual antiplatelet therapy is gender-specific (in order to counteract the increased bleeding risk usually observed in females), but it has a similar clinical outcome irrespective of sex [140].

High percentage of individuals usually experiences antiplatelet therapy resistance that impairs successful prevention of cardiovascular events, and some determinants of resistance to antiplatelet therapy are gender responsive [141–146]. A prospective study on 160 patients with stable coronary heart disease (118 men and 42 women, aged 65.2 ± 7.8 years), indeed, showed a sex-related response to long-term double antiplatelet therapy (75 mg/day aspirin and clopidogrel for three months): female gender was more predisposed to resistance to both aspirin and clopidogrel compared to men [19]. Two main factors may account for the worst responsiveness: women possess (i) greater aggregation capacity, maybe because of higher density of platelet receptors able to bind fibrinogen, and (ii) increased inflammatory status, as highlighted by higher concentrations of the proaggregatory C-reactive protein (CRP) and number of leukocytes and granulocytes.

## 5. Conclusions

**Figure 1:**
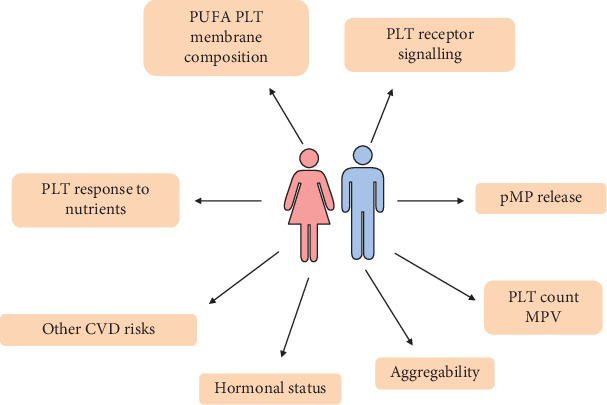
Schematic representation of the main sex-based differences in platelets in relation to cardiovascular disease. See text for details. CVD: cardiovascular disease; MPV: mean platelet volume; PLT: platelet; pMPs: platelet microparticles; PUFA: polyunsaturated fatty acids.

Evidence to date has revealed sex-based differences in CVD prevention, diagnosis, and management. Among modifiable and nonmodifiable risk factors, platelet hyperactivity deserves particular mention, as activation and aggregation of platelets, as well as their interaction with endothelial cells and crosstalk with immune cells, play a major role in the pathophysiology of main cardiovascular events, including stroke, myocardial infarction, and peripheral vascular injury. Moreover, platelet biology is profoundly modulated by several elements, including sex hormones, nutrients, and inflammatory mediators (Figure 1). Consequently, men and women not only display a different platelet functionality but also distinctively respond to common antiplatelet drugs, as well as to specific dietetic habits. In conclusion, full understanding of gender-related differences is the final goal in order to design tailored strategies for preventing and treating platelet-mediated vascular damage.

## Figures and Tables

**Table 1:** Nutritional studies aimed at investigating gender-related differences in platelet responses.

Dietary factors	Experimental protocol	Main findings	Refs
Mediterranean diet (MD)	Population-based cohort study: 6975 males and 7611 females (mean age: 54.2 ± 11.5 yrs) adhering to MD and subdivided into 3 groups according to their PLT count: high-, medium-, and low-PLT count groups (2.5%, 95.6%, and 1.9% of the population, respectively).	In both sexes: PLT count was inversely associated with both MDS and IMI scores. Subjects with very high MD adherence had lower odds of having high-PLT count compared with individuals with poor adherence (OR 50.50; 95% CI: 0.31-0.80 and OR 5 0.73; 95% CI: 0.52-1.02 for MDS and IMI, respectively).	[97]
		In males: the mean PLT count increased with increasing of predicted CVD risk (low CVD risk: 236.5 ± 54.7, medium CVD risk: 239.6 ± 57.1, and high CVD risk: 247.1 ± 58.7; *P* for trend > 0.027 in multivariable analysis of variance).	
		In females: no differences in PLT count within the predicted CVD risk.	

*ω*-3 PUFA	Ex vivo study: PLT isolated from healthy 20 males (33.5 ± 2.1 yrs) and 22 females (35.7 ± 2.5 yrs) preincubated with 1 *μ*M EPA, DHA, or DPA for 6 min at 37°C, before stimulation with 5 *μ*g/mL collagen.	In both sexes: DHA and DPA equally reduced PLT aggregation (36.4% and 33.5% in men and women, respectively). EPA was the most efficacious PUFA (51.7%, *P* < 0.01*vs.* DPA and *P* < 0.004*vs.* DHA).	[105]
		In males: DHA (25.3%) and DPA (21.7%) were less effective, with respect to EPA (48.9%, *P* < 0.002 and *P* < 0.001, respectively).	
		In females: DHA (46.5%), DPA (44.2%), and EPA (54.3%) equally reduced PLT aggregation.	
	Blinded placebo-controlled trial: healthy 15 males (40.1 ± 2.1 yrs old) and 15 females (47.4 ± 1.9 yrs), alternatively receiving a single dose of 2 × 1 g capsules containing either (i) placebo or (ii) EPA-rich oil (providing 1 g EPA with EPA/DHA ratio = 5 : 1) or (iii) DHA-rich oil (providing 1 g DHA with EPA/DHA ratio = 1 : 5). Fasting blood samples collected for PLT aggregation assay at 0, 2, 5, and 24 hrs after supplementation.	In both sexes: EPA- and DHA-rich oils reduced PLT aggregation. EPA was the most effective at 2 (-3.6%, *P* < 0.001), 5 (-8.8%, *P* < 0.001), and 24 (-13.3%, *P* < 0.006) hrs postsupplementation. DHA was inefficacious at 2 and 5 hrs, but equally effective (-11.9%, *P* < 0.016) as EPA at 24 hrs.	[109]
		In males: only EPA reduced PLT aggregation at 2 (-11%, *P* < 0.001), 5 (-10.6%, *P* < 0.003), and 24 (-20.5%, *P* < 0.008) hrs.	
		In females: only DHA reduced PLT aggregation at 24 hrs (-13.7%, *P* < 0.05).	
	Blinded placebo-controlled trial: healthy 15 males (40.1 ± 2.1 yrs) and 15 women (47.4 ± 1.9 yrs), alternatively receiving a single dose of 2 × 1 g capsules containing either (i) placebo or (ii) EPA-rich oil (providing 1 g EPA with EPA/DHA ratio = 5 : 1) or (iii) DHA-rich oil (providing 1 g DHA with EPA/DHA ratio = 1 : 5). Fasting blood samples collected at 0 and 24 hrs after supplementation for PLT aggregation assay and measurement of pMP number and procoagulant activity.	In both sexes: neither oil affected pMP numbers, and only EPA-rich oil produced a decrease in pMP activity (−19.4%, *P* = 0.003).	[110]
		In males: EPA, but not DHA, increased the mean lag time (60 *vs*. 79 sec, +29.5%) and reduced ADP-induced PLT aggregation (−20.5%, *P* = 0.008) and pMP activity (−22%, *P* = 0.008). Inverse relationship between PLT aggregation activity and testosterone levels (*r* = −0.443, *P* = 0.04).	
		In females: DHA, but not EPA, was effective in reducing PLT aggregation (−13.7%), without affecting pMP number and activity.	
	Double-blind, randomized, controlled intervention trial: 79 men and 95 women aged 20–80 yrs receiving six 0.75 g capsules/day providing a total of 1.5 g EPA and 1.77 g DHA (i.e., 3.27 g EPA plus DHA), as TAG, equivalent to the amount in one portion of oily fish and six 0.75 g placebo capsules (high oleic sunflower oil), over 12 months. Fasting blood samples collected at 0 and 12 months after supplementation for lipid composition of platelet membrane.	In both sexes: no differences in basal content of EPA and DHA. Equal dose-dependent increases of EPA and DHA in platelet membrane between male and females after 12-month supplementation.	[131]
		In males: EPA increased in PLT membrane, but without statistical significance.	

Flavanols	Blinded randomized, controlled acute trial: healthy 26 women (23–62 yrs; mean: 38 ± 2.4 yrs) and 16 males (25–65 yrs; mean: 46 ± 3.4 yrs), who acutely ingested 60 g of (i) flavanol-enriched dark chocolate (FDC; 907.4 ± 22.75 mg of flavan-3-ols), (ii) standard dark chocolate (SDC; 382.3 ± 45.20 mg of flavan-3-ols), and (iii) white chocolate (WC; not detectable). Fasting blood collected at 0, 2, and 6 hrs after supplementation for PLT activity assays.	Ex vivo bleeding time In both sexes: *ex vivo* bleeding time increased 6 hrs after consumption of FDC and SDC, but not of WC (*P* = 0.011), in both sexes. In females: *ex vivo* bleeding time increased 6 hrs after the consumption of FDC and SDC, but not with WC (*P* = 0.016). In males: *ex vivo* bleeding time increased 6 hrs after the consumption of FDC and WC (*P* = 0.042).	[122]
		PLT aggregation In both sexes: ADP-induced platelet aggregation reduced at 2 hrs, but not 6 hours, after consumption of FDC and SDC. In males: ADP-induced PLT aggregation was reduced at 2 and 6 hrs after consumption of FDC and SDC (*P* = 0.008 and *P* = 0.020 vs. women). In females: TRAP-induced PLT aggregation was reduced at 2 hrs, but not 6 hours, after consumption of FDC (*P* = 0.010, *P* value for interaction between treatment and gender: *P* = 0.213).	
		PLT activation In both sexes: TRAP-induced fibrinogen binding decreased at 2 and 6 hrs after consumption of FDC and WC (respectively, *P* = 0.014 and *P* = 0.021 vs. SDC). In males: ADP-triggered P-selectin exposure decreased at 2 hrs, but not 6 hrs, after consumption of FDC and WB, but not with SDC (*P* = 0.002). In females: TRAP-induced fibrinogen binding was decreased at 2 hrs, but not 6 hours, after consumption of FDC (*P* = 0.041, *P* value for interaction between treatment and gender: *P* = 0.304).	

Isoflavones	Double-blind, randomized, placebo-controlled study: 29 postmenopausal women (45–60 yrs), who randomly received two daily capsules of a soybean isoflavone extract (23.4 ± 3.4 mg daidzein and 24.1 ± 4.6 mg genistein per capsule) or placebo for 12 weeks. Blood collected at 0 and 12 weeks after supplementation for PLT TxA2 receptor binding assay.	In females: PLT TxA2 receptor density decreased in isoflavone-treated subjects from 181.9 ± 30.9 to 115.2 ± 16.2 fmol/10^8^ PLT (*P* < 0.02 vs. the placebo group). Decrease in TxA2 receptor density inversely correlated with serum concentrations of isoflavones.	[129]
	Double-blind, randomized, placebo-controlled study: healthy 10 men (25.8 ± 1.2 yrs) receiving 60 mg of soy proteins in the form of beverage powder and providing 131 mg of total isoflavones (80.3 mg genistein, 35.6 mg daidzein, and 15.1 mg glycitein) and 10 men (23.9 ± 0.9 yrs), receiving 60 mg of calcium caseinate powder (control), for 28 days. Blood was collected at 0, 28, and 56 days after supplementation for quantification of isoflavone content in plasma and PLT aggregation.	In males: plasma isoflavone content increased after 28 day in the supplementation group (*P* < 0.05*vs.* basal values) and returned to baseline after 56 days (washout period). PLT aggregation was not affected by soy protein supplementation.	[130]

CI: confidence interval; DHA: docosahexaenoic acid; DPA: docosapentaenoic acid; EPA: eicosapentaenoic acid; IMI: Italian Mediterranean Index; MD: Mediterranean diet; MDS: Mediterranean Diet Score; OR: odds ratio; PLT: platelet; pMP: platelet microparticles; TAG: triglycerides; TRAP: thrombin receptor activating peptide; TxA2: thromboxane A2.

## Abbreviations

12-H(P)ETE: 12-Hydro(pero)xyeicosatetraenoic acid
12-LOX: 12-Lipoxygenase
ANOCA: Angina and nonobstructive coronary artery disease
AA: Arachidonic acid
BMI: Body mass index
CRP: C-reactive protein
CVD: Cardiovascular disease
COX-1: Cyclooxygenase 1
DHA: Docosahexaenoic acid
DPA: Docosapentaenoic acid
EPA: Eicosapentaenoic acid
GP: Glycoprotein
PAF: Platelet-activating factor
pMP: Platelet microparticle
PGI_2_: Prostacyclin
PUFA: Polyunsaturated fatty acid
RR: Relative ratio
TxA2: Thromboxane A2
TPA: Thrombin receptor activating peptide
vWF: von Willebrand factor.
